# Elder abuse in the oldest old: prevalence, risk factors and consequences

**DOI:** 10.1007/s00391-021-01945-0

**Published:** 2021-07-30

**Authors:** Thomas Brijoux, Michael Neise, Susanne Zank

**Affiliations:** 1 ceres—cologne center for ethics, rights, economics, and social sciences of health, Universität zu Köln, Albertus Magnus Platz, 50923 Köln, Germany; 2 Lehrstuhl für Rehabilitationswissenschaftliche Gerontologie, Humanwissenschaftliche Fakultät, Universität zu Köln, Köln, Germany; 3 Landschaftsverband Rheinland, Köln, Germany

**Keywords:** Elder abuse, Old age, Representative survey, Elder mistreatment, Loneliness, Elder Abuse, Hochaltrigkeit, Repräsentative Studie, Misshandlung, Einsamkeit

## Abstract

**Background:**

Experiences of abuse in relationships with an expectation of trust are a common phenomenon among older people and is called elder abuse (EA). This can take various forms, such as physical, verbal, emotional, psychological, financial, sexual abuse or neglect. Due to their high vulnerability and difficulties in receiving support, people aged over 80 years old have been pointed out as a group that needs special focus in research.

**Objective:**

Prevalence, risk factors and consequences of EA for different aspects of quality of life are explored among the oldest old.

**Material and methods:**

Computer-assisted personal interviews were conducted in a representative sample of the oldest old in North Rhine-Westphalia (Germany). 988 self-report interviews without third persons present of the NRW80+ study are used to assess EA with the help of the elder abuse and emotional consequences scale (EACS). The EACS describes EA in six dimensions that give a broad understanding of EA.

**Results:**

Prevalence of experiences of EA within the last 12 months was 54.1%. In logistic regression, multimorbidity, lower functioning, age below 90 years, smaller social network size, and aggressive behaviorwere significant risk factors for EA. People experiencing EA showed less life satisfaction and autonomy and increased loneliness and depressive symptoms.

**Conclusion:**

EA is prevalent among the oldest old. Serious consequences of EA on life results can be shown with a broad operationalization of EA. Future research should focus on a deeper understanding of reasons for EA and reflect on the relationship between and the perspectives of perpetrators and victims.

**Supplementary Information:**

The online version of this article (10.1007/s00391-021-01945-0) contains supplementary material, which is available to authorized users.

## Abstract (de)

**Hintergrund:**

Erfahrungen von Gewalt in Beziehungen mit einer Vertrauenserwartung sind ein häufiges Phänomen und werden Elder Abuse (EA) genannt. EA kann in verschiedenen Formen wie physischem, verbalem, emotionalem, psychischem, finanziellem oder sexuellem Missbrauch sowie Vernachlässigung vorkommen. Aufgrund ihrer hohen Verletzbarkeit und erschwerter Hilfeinanspruchnahme im Fall von Viktimisierungserfahrungen wurden Menschen über 80 Jahren als Risikogruppe identifiziert.

**Fragestellung:**

Dieser Artikel untersucht Prävalenz, Risikofaktoren und Auswirkungen von EA auf verschiedene Dimensionen von Lebensqualität in der Hochaltrigkeit.

**Material und Methode:**

Computergestützte persönliche Interviews wurden in einer repräsentativen Stichprobe der Hochaltrigenbevölkerung in Nordrhein-Westfalen durchgeführt. In dieser Analyse werden nur Selbstberichte verwendet, bei denen keine dritten Personen in der Interviewsituation präsent waren. EA wird mit der Elder Abuse and Emotional Consequences Skala (EACS) gemessen. Die EACS unterscheidet zwischen 6 Formen von EA, denen ein breites Verständnis von EA zugrunde liegt.

**Ergebnisse:**

Die Prävalenz von Gewalterfahrungen innerhalb der letzten 12 Monate beträgt 54.1 %. Multimorbidität, Beeinträchtigung in instrumentellen Aktivitäten des täglichen Lebens, Alter unter 90 Jahre, ein kleineres soziales Netzwerk und aggressives Verhalten waren signifikante Risikofaktoren für EA. Menschen, die von EA betroffen waren, zeigten weniger Lebenszufriedenheit und Autonomie, mehr depressive Symptome und waren häufiger von Einsamkeit betroffen.

**Schlussfolgerung:**

Erfahrungen von EA sind in der Hochaltrigenbevölkerung verbreitet. Mit der gewählten weiten Operationalisierung von EA können bedeutende Konsequenzen für die Lebensqualität der von EA Betroffenen aufgezeigt werden. Zukünftige Forschung sollte die Gründe für Gewalthandlungen besser erforschen und die Beziehung wie auch die Perspektiven von Tätern und Opfern berücksichtigen.

## Background

The group of the oldest old (aged over 80 years) is one of the fastest growing populations in Germany [3]. A widespread phenomenon among older people in general (60+ years) is called elder abuse (EA). The most common working definition of EA, which is used in most studies and adopted by the World Health Organization (WHO) describes EA as:

a single or repeated act or lack of appropriate action, occurring within any relationship where there is an expectation of trust which causes harm or distress to an older person. Elder abuse has serious consequences for the health and well-being of older people and can be of various forms: physical, verbal, psychological/emotional, sexual and financial. It can also simply reflect intentional or unintentional neglect [31, p. 1].

The occurrence of EA has been investigated in many national and international studies. Two different meta-analyses reported prevalence in community settings of 10% [13] and 15%, respectively. [33]. Within these two meta-analyses, the range of reported prevalence rates was between 0.8% [20] and 79.7% [26], which indicates heterogeneous definitions and operationalizations of EA that differ between analyzing solely illegal acts and addressing subjective experiences of abuse [8]. In a representative German study, a prevalence rate of 25.6% for psychological aggression and 1.5% for physical violence in community-dwelling people aged between 60 years and 85 years old was reported [8]. In contrast, EA in nursing homes is seldomly explored through the perspective of the victims, which might be due to the high sensitivity of the topic as well as difficult access to and limited communicative abilities of this group [8]. Moreover, a representative prevalence rate of EA in the oldest old (80+ years) is lacking on the international and national levels.

As prevalence rates are missing for the oldest old and consequences of EA are presumably more devastating for them [8], special attention for this group is needed. Firstly, higher need for care, reduction in the size of social networks, higher prevalence rates for dementia, and frailty are reported in this age group [23]. Therefore, psychological forms of violence, like intimidation, paternalism as well as shaming and blaming, should be investigated [21]. Other forms of abuse, like sexual abuse, are rarely reported in old age [33]. Secondly, the victim’s perspective is crucial when EA happens in social relationships with a high dependency between victim and perpetrator. Victim self-reports are of utmost importance when victims have less ability to defend themselves against intentional or unintentional violence, when they have fewer possibilities to reach for help and support, and when their access to social studies is limited [8], all of which is the case for the oldest old.

Risk factors for EA abuse have been explored in two recent systematic reviews [16, 28]. Risk factors for the victims can be categorized into sociodemographic, physical, and psychological aspects [16, 28]. Sociodemographic risk factors with consistent evidence are ethnicity and lower education [28]. Contradicting evidence exists for age > 75 years, gender (female), marital status, higher income, and living alone, which are protective factors in some studies and risk factors in others. Psychological constructs like cognitive impairment, aggressive behavior, loneliness, personality traits (e.g. antisocial personality), self-neglect, and stress-related coping processes are associated with EA [16, 28]. Health-related risk factors comprise various measures of functional impairments like ADL and IADL, frailty, multimorbidity, and incontinence as well as psychiatric illnesses (depression, alcohol abuse, past abuse) [16, 28].

EA affects central aspects of quality of life (QoL) in very old age. In a review of 25 cross-sectional studies, Dong et al. [4] pointed out that EA has a detrimental impact on the psychological well-being of the aged population. In addition, they reported that depression is one of the most prevalent psychological consequences of EA. Recently, Wang and Dong [30] showed that community-dwelling older people who report a greater degree of self-perceived loneliness have a greater probability of having experienced EA, especially psychological forms of abuse. A further aspect of quality of life that is influenced by EA is autonomy, often by use of custodial measures.

The first objective of this study is to estimate the prevalence of various facets of EA in very old age. In a second step, risk factors for experiencing EA are identified. Thirdly, we investigate the effects of EA on central outcomes of QoL in very old age.

## Study design

The NRW80+ dataset is used to examine EA in the oldest old. The sample consists of *n* = 1863 persons over 80 years and includes *n* = 1687 self-report interviews and *n* = 176 proxy interviews. Details about the recruitment process, a description of the proxy interviews, and a general description of the participants of the NRW80+ study are presented elsewhere [10].

The challenges and potentials (CHAPO) model describes the theoretical framework of this study [29]. The definition of EA already implies a connection between life conditions and emotional results which are also contemplated in the broader CHAPO framework. With respect to this model, we have investigated the role of disposable resources, skills, and competencies relating to experiences of EA and linked these experiences to the model-inherent life results depression, loneliness, life satisfaction, and autonomy.

### Variables

The elder abuse and emotional consequences scale (EACS) is used to describe the prevalence of EA within the last 12 months [22]. The EACS is a low-threshold instrument that is designed for use in large representative surveys. It comprises 13 items, describing different actions of EA and their emotional consequences for the victim (e.g. “how often have you experienced that someone raised their voice against you so that you felt upset or insecure”). The frequencies of these actions are graded in five categories from never to very often. We constructed a dichotomous outcome variable for EA: if the interviewed person rated any of the 13 items as seldom or more frequent, this was regarded as EA. The 13 items represent the 6 dimensions: intimidation, shaming and blaming, paternalism, neglect, financial exploitation, and physical abuse.

Sociodemographic risk factors analyzed were gender, age group, nursing home residency, education [32], social network size, and income [2].

Potential psychological risk factors that were analyzed were aggressive and offensive behavior [1] and cognition. Cognition was measured by means of the DemTect [17, 18].

Physical risk factors explored were multimorbidity, frailty, and functional health. The number of treated diseases was used as an indicator of multimorbidity. Frailty was analyzed in accordance with the description by Zimmermann et al. [34], which categorizes people as frail, pre-frail, or non-frail. The instrumental activities of daily living (IADL) subscale of the older Americans resources and services questionnaire was used to describe the functional status [6].

Potential consequences of EA were examined in the form of depressive symptoms, loneliness, autonomy, and life satisfaction. As an indicator for depressive symptoms, we used the short form of the depression in old age scale (DIA-S4) [12]. The DIA-S4 is a screening tool for depression consisting of four questions that are part of the DIA‑S [11]. Loneliness [5], autonomy and life satisfaction [19] were measured with one item that has been established in the socioeconomic panel and the European social survey. A more detailed description of the used variables can be obtained in Supplement 1.

### Sample and bias

**Fig. 1:**
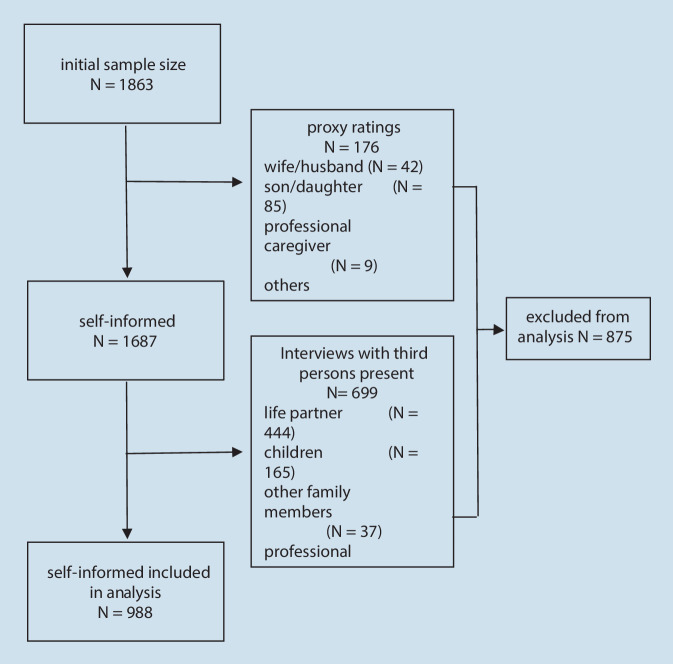
Self-report interviews, proxy interviews, interviews conducted in the presence of others

With use of survey weights, the NRW80+ sample is representative in relation to age, sex, and nursing home status. Detailed analyses that are published elsewhere [22] point out limitations in interviews in which third persons were present or which were conducted with proxy informants. In this article, only self-ratings from interviews in which the interviewee and the interviewer were alone were analyzed. Therefore, 176 proxy ratings and 699 interviews in which third persons were present were excluded from this analysis. The resulting sample consisted of 988 cases (see Fig. 1).

The exclusion of proxy interviews and interviews where third persons were present lead to a selective and biased subsample. This bias was reduced by a recalculation of sample weights via logistic regression [7] that gives more weight to underrepresented groups. Consequences of weighting the sample are shown in Supplement 2.

### Statistical methods

Frequency of EA and its dimensions were analyzed by descriptive statistics. Confidence intervals for binary variables are 95% Clopper-Pearson intervals. Odds ratios and Cohens D are presented as measures of effect size. Logistic regression is used to examine risk factors of EA. Tests for multicollinearity show no problems. Multiple imputation [15] was used to address missing values, with Rubins formula [25] for the calculation of standard deviations, 20 datasets were imputed [9]. The Holm-Bonferroni for multiple tests is used [14]. Calculations were carried out by means of SPSS (IBM Corp. Released 2020. IBM SPSS Statistics for Windows, Version 27.0. Armonk, NY, USA) Version 27 and Microsoft Excel 365.

## Results

### Prevalence of elder abuse

About half of the sample (*N* = 534; 54.1%; Confidence Interval (CI): 51.0–57.2%) have experienced a form of EA at least once during the last 12 months. The most frequent form of EA is intimidation with a prevalence of 39.2% (CI: 36.2–42.3%). Other frequent forms are paternalism (32.6%; CI: 29.8–35.6%), neglect (27.0%, CI: 24.4–29.9%), and shaming and blaming (23.1%, CI: 20.6–25.9%). Financial exploitation (10.9%, CI: 9.1–13.0%) and physical behavior (8.8%, CI: 7.2–10.8%) occurred less often. Frequent experience of EA (i.e. often or very often) is rare (i.e. less than 4% of the sample) in all facets of EA (Table 1).

**Table 1:** Prevalence and frequency of different EA dimensions

EA-Dimension	Never (in %)	Seldom (in %)	Sometimes (in %)	Often (in %)	Very often (in %)
Intimidation	60.8	26.6	10.8	1.4	0.5
Shaming and blaming	76.9	21	1.8	0.3	0
Paternalism	67.4	22.4	7.7	2.2	0.4
Neglect	73	17.8	5.8	2.2	1.2
Financial exploitation	89.1	6.8	2.8	1	0.4
Physical behavior	91.2	5.3	3	0.2	0.3

### Risk factors for elder abuse

Multivariate logistic regression identifies higher multimorbidity (OR: 1.13, *p* < 0.001), social network size (OR: 0.75, *p* < 0.001), and higher levels of aggression of the victim (OR: 2.53, *p* < 0.001) as significant risk factors for EA. No significant influence could be found for cognitive status, education, gender, income, IADL, age group, and frailty. The mean of Nagelkerkes R^2^ over all imputed datasets is 0.16. All regression coefficients and their respective odds ratios and confidence intervals are shown in Supplement 3.

### Quality of life

People who are affected by EA show worse values in all analyzed QoL outcomes. They show more depressive symptoms (1.14 vs. 0.69, *p* < 0.001), perceive more loneliness (1.49 vs. 1.32, *p* < 0.001) and lower autonomy (3.41 vs. 3.63, *p* < 0.001), and have a lower life satisfaction (7.67 vs. 8.25, *p* < 0.001) (Table 2). Effect sizes range between small and medium effects.

**Table 2:** Dimensions of quality of life and EA

		Mean	SD	T	*p*-value^a^	Cohens D
Depression	No EA (*N* = 453)	0.69	1.00	6.32	< 0.001***	0.43
	EA (*N* = 535)	1.14	1.17			
Loneliness	No EA (*N* = 453)	1.32	0.62	−3.66	< 0.001***	0.20
	EA (*N* = 535)	1.49	0.82			
Autonomy	No EA (*N* = 453)	3.63	0.64	−4.73	< 0.001***	−0.26
	EA (*N* = 535)	3.41	0.81			
Life satisfaction	No EA (*N* = 453)	8.25	1.73	−4.82	< 0.001***	−0.42
	EA (*N* = 535)	7.67	2.02			

*p 0.05, **p < 0.01, ***p < 0.001

^a^After the Holm-Bonferroni correction all *p*-values remain significant on the 5% level

## Discussion

During the last 12 months, 54.1% of persons aged 80 years and older have experienced some kind of EA. This prevalence is higher compared to other studies for the elderly population in Germany [8]; however, the comparison of different prevalences of EA can be misleading when the difference in operationalizations is not accounted for. In this sample, the low-threshold approach in the wording of the questions and the emphasis of the EACS on emotional forms of abuse are expected to have contributed to higher prevalence rates. The higher age of the informants, which is associated with other risk factors like multimorbidity, also contributes to the higher prevalence. In old age, emotional and psychological abuse are the most common forms of abuse, which resembles the results of known the meta-analysis and reviews [13, 27, 33] in which emotional abuse was also identified as the most frequent form of EA in younger populations. Even though many very old people experience EA, it is nevertheless a rare event for most of them. In each dimension, less than 4% of the people are affected “often” or “very often” by EA.

In the regression model, multimorbidity, and the victim’s tendency for aggressive interactions are significant risk factors for EA, while a greater social network is a protective factor. An influence of cognition, nursing home status, IADL, education, gender, age group and income could not be shown in the multivariate logistic regression model. While evidence for the effect of gender, education, age group and income has already been inconsistent in existing literature [16, 28], reasons for the missing effect of nursing home status and cognition are not obvious. In a subsequent bivariate sensitivity analysis of our data, nursing home status has shown a significant association with EA, but this effect was moderated by cognition, functional health, and multimorbidity, which are also more common in nursing homes. Therefore, the effect was attenuated in the full model. The same applies to the constructs related to physical health, where IADL is not significant when multimorbidity is included in the model.

Risk factors for the abusers, like caregiver burden, especially for caregivers of people with dementia or substance abuse [16], cannot be investigated in this article. Likewise, risk factors lying within the victim-perpetrator relationship cannot be explored in this article. Therefore, the overall low amount of explained variance was expected.

On the basis of the CHAPO model, life events, such as experiences of EA, are associated with more generalized life results. In our sample and understanding, EA is associated with higher depression, increased loneliness, a reduction of autonomy, and life satisfaction. For depression, loneliness, and life satisfaction, the observed effect sizes are even higher than reported effect sizes in other populations [4, 24], supporting the proposition that the impact of EA is more severe for vulnerable groups [8]. Autonomy and loneliness are also significantly associated with EA. These two life results are more strongly affected by interactions with others, i.e. victim-perpetrator interaction in the case of EA. For a better understanding of underlying processes, the inclusion of the perspective of potential perpetrators in studies seems necessary. This could be done in future qualitative analyses but is not possible within the NRW80+ dataset. For loneliness, it might also be true that old people who have a smaller social network may feel more alone when they experience abuse in one or more of their few relationships. Causality might also work in the other direction, meaning that loneliness makes old people easier to be taken advantage of.

Based on associations found between aspects of QoL and EA, we conclude that our choice of a broad understanding and low-threshold operationalization of EA leads to results that are relevant for the oldest old and should be pursued in future research, especially in the context of quality of life.

Two limitations of the current study should be considered. Firstly, the exclusion of proxy interviews limits the representativity of the sample. The resulting bias has been addressed by a recalculation of sample weights that gives more importance to cases which are underrepresented; however, a complete elimination of this bias is not possible. Nevertheless, compared to surveys that do not include people with dementia and focus entirely on community-dwelling older adults, the use of adapted sample weights represents an improvement. Secondly, testing causality was not possible within this cross-sectional dataset and results should be replicated. As control group designs are no ethically possible alternative, longitudinal studies need to be performed.

## Practical implications

- Experiences of EA are a common phenomenon in the group of older people and refer most often to psychological abuse; however, for most of the affected individuals, these actions of EA remain rare events.
- EA is associated with higher depression and loneliness as well as lower life satisfaction and autonomy.
- Longitudinal surveys are needed to unveil mechanisms that lead to EA.
- To analyze mechanisms of EA, studies are needed that reflect on the relationship between perpetrators and victims.

## Supplementary Information

Supplement 1: Risk factors of Elder abuse in logistic regression
