# Supplementary material for: Elder abuse in the oldest old: prevalence, risk factors and consequences
Source: Z Gerontol Geriatr. 2021 Jul 30;54(Suppl 2):132–7. doi: 10.1007/s00391-021-01945-0 (PMC8551100; doi:10.1007/s00391-021-01945-0)
Supplement: Supplementary file 1 — Supplement 1: Risk factors of Elder abuse in logistic regression [file 391_2021_1945_MOESM1_ESM.docx]

Supplement 1

Variable description:

The Elder Abuse and Emotional Consequences Scale (EACS) is used to describe the prevalence of EA within the last twelve months [23]. The EACS is a low-threshold instrument that is designed for use in large representative surveys. It comprises 13 items, describing different actions of EA and their emotional consequences for the victim (e.g. “How often have you experienced that someone raised their voice against you so that you felt upset or insecure”). The frequencies of these actions are graded in five categories from never to very often. Cronbach’s Alpha for the whole scale is 0.87. We constructed a dichotomous outcome variable for EA: If the interviewed person rated any of the 13 items as seldom or more frequent, this was regarded as EA. The 13 items represent the six dimensions: Intimidation, shaming and blaming, paternalism, neglect, financial exploitation, and physical abuse. The intention is to link the construct closely to feelings of the victims and not only to actions of potential perpetrators. Therefore, the items include aspects like felt neglect and or not limited to any specific settings like nursing homes.

Sociodemographic risk factors analyzed were gender, age group, nursing home residency, education, social network size, and income. Education was assessed in three categories established by the German Ageing study [33]. Income was measured as household net income and is analysed in six categories that were established in the household budget surveys [2].

Potential psychological risk factors that were analysed were aggressive and offensive behaviour and cognition. Three items from the “too aggressive” subscale of the Inventory of Interpersonal Problems were used to measure aggressiveness [1].

Cognition was measured by means of the DemTect. The DemTect is a screening instrument for early dementia and mild cognitive impairment with good classification for dementia (100% sensitivity and 92% specificity) of and mild cognitive impairment (80% sensitivity and 92% specificity) [18]. It provides age-specific scores for the oldest old [19]. The Cut-Off scores 13 and 9 were used to differentiate between normal ageing, mild cognitive impairment and dementia [18].

Physical risk factors explored were multimorbidity, frailty, and functional health. The number of treated diseases was used as an indicator of multimorbidity. Frailty was analysed in accordance with the description by Zimmermann et al. [35], which categorizes people as frail, pre-frail, or non-frail. The “instrumental activities of daily living” (IADL) subscale of the Older Americans Resources and Services Questionnaire was used to describe the functional status [7].

Potential consequences of EA were examined in the form of depressive symptoms, loneliness, autonomy, and life satisfaction. As an indicator for depressive symptoms, we used the short form of the Depression in old Age Scale (DIA-S4) [13]. The DIA-S4 is a screening tool for depression consisting of four questions that are part of the Depression in old Age Scale [12]. Loneliness [6], autonomy and life satisfaction [20] were measured with one-item that have been established in the socio-economic Panel and the European social survey.

Loneliness was measured with the item “How often have you felt lonely during the last week?” which was also used in the seventh wave of the European Social Survey [6] and could be answered in four points. A single item was used to measure autonomy (“Do you arrange your life according to your own ideas?”) on a four-point scale. Life satisfaction was measured with an eleven-point item ranging from zero to ten (“All in all, how satisfied are you currently with your life?”), that has been used similarly in the socio-economic panel [20].

Sample description

| Construct | Category | NRW 80+ Full Sample (N = 1863)^1^ | EA Sample (N = 988) (using survey weights) ^2^ | EA Sample (analysis weights) (N = 988)^3^ |
| --- | --- | --- | --- | --- |
| Gender | Male | 36.3% | 30.2% | 35.6% |
|  | Female | 63.7% | 69.8% | 64.4% |
|  |  |  |  |  |
| Cognition | Healthy | 68.1% | 75.8% | 71.1% |
|  | Mild cognitive Impairment | 15.5% | 15.7% | 15.8% |
|  | Dementia | 16.4% | 8.5% | 13.1% |
|  |  |  |  |  |
| Age group | 80-84 | 54.3% | 54.1% | 55.2 % |
|  | 85-89 | 30.8% | 33.0% | 31.4% |
|  | 90+ | 14.9% | 12.9% | 13.3% |
|  |  |  |  |  |
| Education | Low | 27.8% | 24.8% | 28.5% |
|  | Middle | 53.0% | 53.8% | 51.4% |
|  | High | 17.8% | 21.3% | 20.1% |
|  |  |  |  |  |
| Household Income | below 1300 € | 16.4% | 18.4% | 25.8% |
|  | 1300 € - 1700 € | 14.1% | 15.6% | 20.7% |
|  | 1700 € - 2600 € | 23.5% | 22.7% | 29.8% |
|  | 2600 € - 3600 € | 11.8% | 11.4% | 14.4% |
|  | 3600 € - 5000 € | 4.4% | 4.4% | 6.0% |
|  | 5000 € - 18000 € | 2.9% | 2.6% | 3.3% |
|  |  |  |  |  |
| Nursing Home | No | 86.1% | 86.5% | 86.7% |
|  | Yes | 13.9% | 13.5% | 13.3% |
|  |  |  |  |  |
| Frailty | Non-Frail (Ref.) | 25.2% | 31.2% | 20.6% |
|  | Pre-Frail | 59.0% | 57.0% | 42.2% |
|  | Frail | 15.8% | 11.8% | 11.7% |
|  |  |  |  |  |
| IADL |  | 1.4 (0.69) | 1.58 (0.54) | 1.44 (0.62) |
|  |  |  |  |  |
| Multimorbidity | #Diseases (SD) | 3.61 (2.36) | 3.36 (2.30) | 3.42 (2.32) |
|  |  |  |  |  |
| Loneliness |  | 1.35 (0.67) | 1.38 (0.70) | 1.41 (0.74) |
|  |  |  |  |  |
| Depression |  | 0.93 (1.13) | 0.87 (1.12) | 0.91 (1.12) |
|  |  |  |  |  |
| Life Satisfaction |  | 7.78 (2.02) | 7.98 (1.89) | 7.95 (2.10) |
|  |  |  |  |  |
| Autonomy |  | 3.43 (0.86) | 3.59 (0.69) | 3.52 (0.83) |

1 Sample description of the NRW 80+ dataset using original weights. 2 Sample after exclusion of proxy interviews and interviews with third persons present using original weights. 3 Sample as analysed using new sample weights**.**

Supplement 2 Risk factors for EA

|  |  |  | Overall Model | | |
| --- | --- | --- | --- | --- | --- |
| Variable |  | β | OR | OR CI | p-value^1^ |
| Age group | 80 - 84 (Ref.) (n = 389) |  |  |  |  |
|  | 85 – 89 (n = 363) | -0.17 | 0.85 | 0.62 - 1.16 | 0.29 |
|  | 90+ (n = 236) | -0.49 | 0.61 | 0.39 - 0.96 | 0.03* |
| Gender | Men (Ref.) (n = 440) |  |  |  |  |
|  | Women (n = 548) | 0.12 | 1.13 | 0.81 - 1.55 | 0.48 |
| Education | Low (Ref.) (n = 226) |  |  |  |  |
|  | Medium (n = 535) | 0.34 | 1.40 | 0.96 - 2.05 | 0.08 |
|  | High (n = 228) | 0.44 | 1.55 | 0.86 - 2.81 | 0.15 |
| Nursing Home | No (Ref.) (n = 873) |  |  |  |  |
|  | Yes (n = 115) | 0.34 | 1.41 | 0.83 - 2.4 | 0.21 |
| Household Income | below 1300 € (Ref.) (n = 210) |  |  |  |  |
|  | 1300 € - 1700 € (n = 212) | -0.09 | 0.92 | 0.56 - 1.5 | 0.73 |
|  | 1700 € - 2600 € (n = 275) | 0.42 | 1.52 | 0.96 - 2.4 | 0.08 |
|  | 2600 € - 3600 € (n = 147) | 0.44 | 1.56 | 0.85 - 2.88 | 0.15 |
|  | 3600 € - 5000 € (n = 72) | 0.24 | 1.27 | 0.49 – 3.29 | 0.62 |
|  | 5000 € - 18000 € (n = 72) | 0.28 | 1.32 | 0.40 – 4.33 | 0.64 |
| Social network | No. of persons | -0.29 | 0.75 | 0.66 – 0.85 | <0.001*** |
| Personality | Aggression | 0.93 | 2.53 | 1.83 - 3.49 | <0.001*** |
| Cognition | Normal (Ref.) (n = 723) |  |  |  |  |
|  | MCI (n = 165) | -0.07 | 0.94 | 0.61 - 1.45 | 0.77 |
|  | Dementia (n = 100) | -0.06 | 0.94 | 0.54 - 1.65 | 0.83 |
| Multimorbidity | No. of Diseases | 0.13 | 1.13 | 1.06 - 1.21 | <0.001*** |
| Frailty | Non-Frail (Ref.) (n = 281) |  |  |  |  |
|  | Pre-Frail (n = 564) | 0.21 | 1.23 | 0.8 - 1.90 | 0.34 |
|  | Frail (n = 144) | 0.52 | 1.68 | 0.89 - 3.2 | 0.11 |
| Functional Health | IADL | -0.37 | 0.69 | 0.49 - 0.97 | 0.03* |
| Constant |  | -1.38 | 0.25 | 0.09 – 0.71 | <0.01** |
| Overall R^2^ |  |  |  |  | 0.16 |

*p.0.05, **p< 0.01, ***p< 0.001

^1^The shown p-values are not adjusted for multiple testing. Using the Holm-Bonferroni adjustement only social network, personality und multimorbidity are significant at the 5% level.
